# Health-related quality of life following salvage radical prostatectomy for recurrent prostate cancer after radiotherapy or focal therapy

**DOI:** 10.1007/s00345-024-04945-y

**Published:** 2024-04-18

**Authors:** Severin Rodler, Dina Danninger, Lennert Eismann, Philipp Maximilian Kazmierczak, Friedrich Jokisch, Minglun Li, Armin Becker, Alexander Kretschmer, Christian Stief, Thilo Westhofen

**Affiliations:** 1 Department of Urology, LMU University Hospital, Marchioninistr. 15, 81377 Munich, Germany; 2 Department of Radiology, LMU University Hospital, Munich, Germany; 3 Department of Radiotherapy, Klinikum Lüneburg, Lüneburg, Germany

**Keywords:** Quality of life, Salvage radical prostatectomy, Long-term outcome

## Abstract

**Background:**

Salvage radical prostatectomy (sRP) is an important treatment option for patients with recurrent prostate cancer (PCa) after radiotherapy (RT) or focal therapy (FT). However, health-related quality of life (HRQOL) after sRP depending on the primary treatment is understudied.

**Methods:**

Patients who underwent Salvage RP for recurrent PCa were analyzed. The primary outcome of this study was HRQOL assessed by the quality-of-life questionnaire (QLQ)-C30 and its prostate specific QLQ-PR25 add-on. Secondary outcomes were functional outcome parameters (erectile function, continence) and biochemical recurrence-free survival (BRFS). Statistical analyses employed the chi-square test, Mann–Whitney *U* test, and Kaplan–Meier method, with a *p* value < 0.05 denoting significance.

**Results:**

37 patients with RT as primary treatment (RT-sRP) and 22 patients with focal therapy prior sRP (FT-sRP) were analyzed. Mean global health score was not significantly different preoperatively (71.9 vs. 67.3, *p* = 0.89) as well as after a median of 32 months follow-up (54.9 vs. 50.6, *p* = 0.63) with impaired HRQOL after sRP in both groups. Baseline erectile dysfunction was more prevalent in the RT-sRP group (mean IIEF-5: 5.0) than in the FT-sRP group (mean IIEF-5: 8.5, *p* = 0.037). No differences were observed at follow-up for erectile function (IIEF-5-Score: 0.5 vs 2.5, *p* = 0.199) and continence (continence rate: 48.4% vs 52.9% (*p* = 0.763) between the RT-sRP and FT-sRP group. 5-year-BRFS was 60% (RT-sRP) and 68% (FT-sRP, *p* = 0.849).

**Conclusions:**

sRP impacts HRQOL in patients with PCa after RT and FT with no significant differences. Comparison with HRQOL and BRFS of treatment alternatives is paramount to counsel patients for appropriate treatments.

**Supplementary Information:**

The online version contains supplementary material available at 10.1007/s00345-024-04945-y.

## Introduction

Salvage radical prostatectomy (sRP) is an established treatment option for patients with recurrent prostate cancer following primary treatment of the prostate, by radiotherapy (RT) or focal therapy (FT). Salvage RP provides sufficient long-term cancer control regardless of the primary treatment modality, with 5-year cancer-specific survival rates up to 95% [1, 2]. Yet only few patients with local prostate cancer recurrence after non-surgical primary treatment will receive sRP [3]. This might be due to inherent technical challenges, considerably high complication rates and poor functional results, with earlier studies reporting urinary incontinence rates up to 90% following sRP [4]. More recent studies however have reported better functional results for patients treated with sRP with continence rates up to 90% [5, 6]. In direct comparison of functional results depending on primary treatment modality, higher continence rates following sRP are reported after focal therapy [7].

However, evidence on patient-reported outcome measures (PROMs) following sRP is scarce, with unknown impact of the primary treatment modality on health-related quality of life (HRQOL). With growing recognition of the guiding role of patients’ perspectives, HRQOL has gained importance in clinical decision making [8]. Therefore, driven by this paucity of data we conducted this first comparative analysis of PROMS from a contemporary sRP cohort. We hereby aimed to assess the impact of primary treatment modality on long-term HRQOL-outcomes following sRP.

## Patients and methods

### Patient population, study design, and data assessment

After approval by a local institutional ethics committee (#20-1022), patients from a prospective institutional database who underwent sRP between January 2008 and December 2019 were identified. Surgical techniques in our department have been described before and rely mainly on open approaches for salvage treatment [9]. Decision for nerve-sparing was performed intraoperatively. Patients were stratified by primary treatment modality: External beam radiotherapy [= radiotherapy (RT)] or focal therapy [High-Intensity Focused Ultrasound (HIFU), vascular-targeted photodynamic therapy (VTP)]. A flow chart illustrating the patient selection is provided in supplementary Fig. 1.

### Outcomes

Primary endpoint was HRQOL based on validated questionnaires. Assessment of HRQOL was performed using the standardized European Organization for Research and Treatment of Cancer (EORTC) quality of life questionnaire (QLQ)-C30 and its prostate specific QLQ-PR25 add-on [10]. According to established cut-off values, “good general HRQOL” was defined as a global health status (GHS) of ≥ 70 [11].

Secondary endpoints encompassed functional outcome parameters, biochemical recurrence-free survival (BRFS). Urinary continence was assessed by the International Consultation of Urinary Incontinence questionnaire in its short-form (ICIQ-SF) [12], and daily pad usage. Continence recovery was defined by use of up to one (dry) security pad per 24 h. Erectile function was assessed with the simplified International Index on Erectile Function (IIEF-5) questionnaire [13]. According to institutional standards, questionnaires were handed out to patients 1 to 3 days prior RP. BRFS was defined as the time from RP to biochemical recurrence defined as two consecutive PSA values ≥ 0.2 ng/ml after sRP following current guidelines [14].

### Follow-up

Follow-up of eligible patients was performed at 3 months after surgery (postop), followed by annually intervals thereafter. Hereby, validated questionnaires have been sent to eligible patients via mail. Oncological outcome information was retrieved directly from patients, referring urologists and primary physicians.

### Statistical analysis

Statistical analysis was performed using MedCalc Statistical Software version 20.011 (MedCalc Software, Ostend, Belgium). To test for normal distribution of variables, Shapiro–Wilk test was performed. For descriptive statistics, median and means were used to present continuous variables and percentages or absolute numbers to present non-continuous variables. Chi-square test and Mann–Whitney *U* test were applied for univariate analyses of categorical variables and continuous variables, respectively. Multivariable binary logistic regression was used to identify predictive features for “good general HRQOL” defined as GHS ≥ 70. Spearman rank correlation was applied to identify the relationship between the time interval from primary treatment to sRP and long-term general HRQOL. Multivariable linear logistic regression was used to identify independent predictors for improved long-term HRQOL. Survival and continence recovery probabilities were estimated applying Kaplan–Meier method and compared using log-rank test. A *p* value of < 0.05 was considered statistically significant.

## Results

### Baseline characteristics of study cohort

Between January 2008 and December 2022, 59 patients were identified that matched the inclusion criteria and had complete follow-up. Median follow-up was 32 months. 37 patients had undergone RT as primary treatment (RT-sRP), whereas 22 patients had undergone focal therapy prior sRP (FT-sRP). Median age for the RT-sRP group was 69 (IQR: 64.5; 72.0) years and for the FT-sRP group 65.5 (IQR: 57.8, 70.3, *p* = 0.046). All other baseline characteristics were not significantly different across the two groups. For further baseline characteristics, please refer to Table 1.

**Table 1:** Patient characteristics

	Percutaneous RT	Focal therapy	*p*
No. of patients	37	22	
Age, yrs [median, IQR]	69.0 [64.5, 72.0]	65.5 [57.8, 70.3]	***0.046***
BMI, kg/m^2^ [median, IQR]	27.0 [24.7, 30.7]	26.2 [24.2, 29.1]	0.335
PSA preop., ng/ml [median, IQR]	5.4 [3.0, 9.6]	5.3 [3.4, 8.6]	0.662
Prostate volume, ml [median, IQR]	38.0 [34.0, 50.0]	37.5 [27.0, 43.3]	0.316
Time between primary treatment to sRP, mo [median, IQR]	60 [32, 99]	38 [17, 89]	0.218
Gleason score [*n* (%)]			
≤ 6	1 (2.7)	3 (13.6)	0.253
7	17 (45.9)	10 (45.5)	
≥ 8	19 (51.4)	9 (40.9)	
pT stage [*n* (%)]			
≤ pT2a	16 (43.2)	10 (45.5)	0.984
pT3a	9 (24.3)	5 (22.7)	
≥ pT3b	12 (32.4)	7 (31.8)	
Charlson comorbidity index [*n* (%)]			
0	5 (13.6)	7 (31.8)	0.288
1	16 (43.2)	8 (36.4)	
≥ 1	16 (43.2)	7 (31.8)	
Surgical approach [*n* (%)]			
Open	36 (97.3)	22 (100.0)	1.000
Robotic-assisted	1 (2.7)	0 (0.0)	
Positive surgical margin [*n* (%)]	11 (29.7)	8 (36.4)	0.389
Lymph node involvement [*n* (%)]	8 (21.6)	1 (4.5)	0.078
Nerve sparing [*n* (%)]	5 (13.6)	3 (13.6)	1.000

*P*-values below 0.05 were deemed significant and are depicted in bolditalics

### Primary treatment modality and HRQOL

Detailed preoperative and postoperative results on general HRQOL and QLQ-C30 subscales are outlined in Table 2. In summary, the preoperative mean GHS score (71.9 vs. 67.3) did not significantly differ between sub-cohorts with comparable rates of patients reporting “good general HRQOL” (50.0 vs. 46.2%; *p* = 0.837). In line functional- and symptom subscales did not significantly differ between both cohorts (*p*-range: 0.1–1.0). Similarly, patients did not report significantly different general HRQOL after median follow-up of 32 months, with comparable mean GHS scores (54.9 vs. 50.6; *p* = 0.63) and comparable rates of patients with good general HRQOL (27.3 vs. 23.1%; *p* = 0.784). Consistently, functional as well as symptom subscales were comparable for both cohorts (*p*-range: 0.24–0.94). Furthermore, QLQ-PR25-subscales were similar between both cohorts (*p*-range 0.20–0.84) (Table 2). The largest net decline in subscales of the QLQ-C30 questionnaire was seen for social functioning (RT-sRP: − 40, FT-sRP: − 27.2) as well as role functioning (RT-sRP: − 38.5, FT-sRP: − 17.1). Fatigue (21.2) and dyspnea (20.5) were predominantly improved in the FT-sRP group, while fatigue (3.5) and dyspnea (− 2.8) revealed only minor changes in the RT-sRP group (supplementary Fig. 2).

**Table 2:** Global health status and functional outcomes

	T0			Follow-up		
	Percutaneous RT	Focal therapy	*p*	Percutaneous RT	Focal therapy	*p*
Erectile function						
IIEF-5 score [mean, SD]	5.0 (8.6)	8.5 (9.2)	***0.037***	0.5 (1.2)	2.5 (4.6)	0.199
IIEF-5 score 18 or more [%]	16.0	21.1	0.667	10.0	14.3	0.703
Urinary continence						
ICIQ-SF score [mean, SD]	2.0 (4.5)	3.7 (5.4)	0.199	13.6 (5.1)	12.3 (6.8)	0.696
Daily pad use [mean, SD]	n.a	n.a	n.a	4.1 (2.9)	3.9 (2.6)	0.940
Urinary continence [%]	87.5	66.7	0.117	48.4	52.9	0.763
Health-related quality of life						
EORTC QLQ-C30						
Symptom scale						
Dyspnea	25 (33.3)	12.8 (21.7)	0.35	22.2 (29.6)	33.3 (35.1)	0.43
Pain	13.5 (26.0)	16.7 (29.7)	0.94	29.2 (39.0)	26.7 (27.4)	0.86
Fatigue	21.5 (23.8)	18.8 (22.4)	0.75	25.0 (28.9)	40.0 (27.8)	0.24
Insomnia	25 (33.3)	17.9 (25.9)	0.64	39.4 (44.3)	36.7 (33.1)	0.94
Appetite loss	4.2 (11.4)	5.1 (18.5)	0.74	8.3 (15.1)	20.0 (32.2)	0.40
Nausea/vomiting	0.0 (0.0)	0.0 (0.0)	1.00	5.6 (14.8)	5.0 (8.1)	0.56
Constipation	10.4 (29.1)	15.4 (17.3)	0.10	25.0 (28.9)	26.7 (34.4)	0.94
Diarrhea	12.5 (20.6)	20.5 (21.7)	0.26	16.7 (22.5)	20.0 (23.3)	0.71
Financial difficulty scale	6.3 (13.4)	10.3 (16.0)	0.46	16.7 (26.6)	16.7 (32.4)	0.87
Functioning scale						
Physical	85.8 (21.3)	92.8 (10.7)	0.66	75.6 (29.5)	82.0 (14.4)	0.97
Role	88.5 (20.8)	82.1 (30.0)	0.58	50.0 (38.3)	65.0 (41.2)	0.29
Cognitive	93.8 (10.3)	92.3 (14.6)	0.98	68.1 (30.5)	76.7 (30.6)	0.33
Emotional	77.1 (16.5)	67.3 (29.7)	0.46	63.2 (29.0)	48.3 (34.2)	0.29
Social	84.4 (16.6)	70.5 (24.7)	0.12	44.4 (37.2)	43.3 (38.7)	0.92
Global health status	71.9 (19.0)	67.3 (27.7)	0.89	54.9 (25.3)	50.6 (23.2)	0.63
Global health status ≥ 70 (%)	50.0	46.2	0.837	27.3	23.1	0.784
EORTC QLQ-PR25						
Urinary symptoms				41.3 (32.9)	46.4 (27.4)	0.84
Incontinence aid				79.2 (39.6)	77.8 (34.4)	0.87
Bowel symptoms				10.8 (18.9)	7.3 (7.0)	0.74
Treatment symptoms				16.7 (13.9)	22.2 (13.3)	0.53
Sexually active				23.3 (25.1)	41.7 (32.1)	0.20
Sexual functioning				46.7 (17.3)	61.1 (34.7)	0.65

*P*-values below 0.05 were deemed significant and are depicted in bolditalics

### Predictors of HRQOL

In multivariable logistic regression analysis of the primary endpoint “good general HRQOL”, neither primary treatment type (*p* = 0.43), time between primary treatment and sRP (*p* = 0.22), good erectile function at baseline (*p* = 0.93), continence recovery (*p* = 0.59), biochemical recurrence (*p* = 0.21), nor increased CCI (*p* = 0.49) could be identified as independent predictors for “good general HRQOL”. Details of the multivariable logistic regression analysis are displayed in Table 3.

**Table 3:** Predictors of good HRQOL after salvage radical prostatectomy

Multivariate logistic regression for good HRQOL at max follow-up					
Predictive feature for good HRQOL	Regression coefficient	Odds ratio	95% CI		*p* value
			Lower	Upper	
Primary treatment type	− 1.631	0.196	0.00	10.81	0.43
Time between primary treatment to sRP	0.025	1.025	0.99	1.07	0.22
IIEF-5 18 or more [yes vs. no]	− 0.134	0.874	0.04	21.20	0.93
Continence recovery [yes vs. no]	− 1.148	0.317	0.01	19.70	0.59
pT stage	1.863	6.441	0.41	10.10	0.18
Gleason-grade	− 1.850	0.157	0.01	2.66	0.20
Lymph node involvement	3.820	4.563	0.15	13.85	0.19
pre-OP PSA	− 2.577	0.076	0.00	3.48	0.19
Biochemical recurrence [yes vs. no]	− 2.449	0.086	0.00	3.95	0.21
Charlson comorbidity Index	− 1.237	0.290	0.01	9.96	0.49

**Fig. 1:**
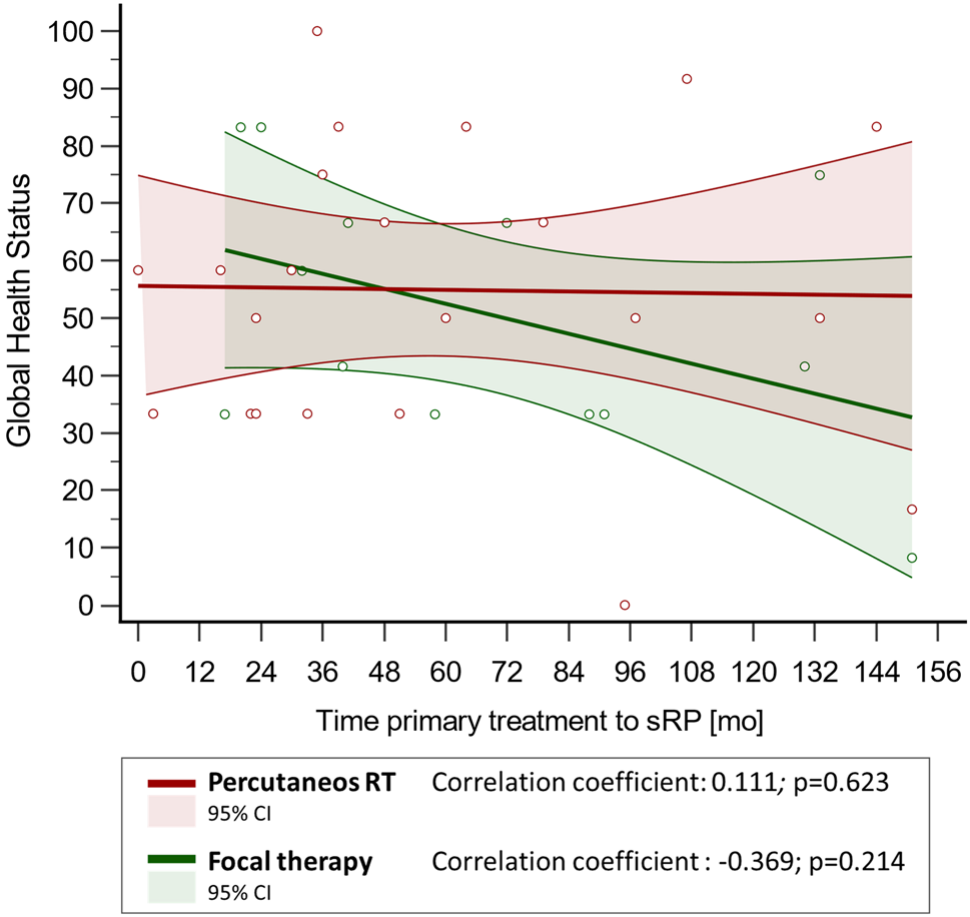
Time-to-salvage prostatectomy and health-related Quality of Life

Spearman’s rank correlation revealed no correlation between time from primary treatment to sRP and long-term general HRQOL (GHS score) equally for the RT-sRP-cohort (*p* = 0.623) and for the FT-sRP-cohort (*p* = 0.214) (Fig. 1).

In multivariable linear regression analysis, a longer time interval between primary treatment and sRP could not be identified as an independent predictor for altered general HRQOL (*p* = 0.788). A reduced postoperative ICIQ-SF-score however could be identified as an independent predictor for improved general HRQOL (*p* = 0.021). The multivariable linear regression analysis is summarized in supplementary Table 1.

### Primary treatment modality and functional outcomes

At baseline, patients in the RT-sRP group reported significantly worse erectile function (mean IIEF-5: 5.0) than in the FT-sRP group (mean IIEF-5: 8.5, *p* = 0.037). Urinary continence was not significantly different at baseline with mean ICIQ-SF-scores of 2.0 vs. 3.7 (*p* = 0.199) and a urinary continence rate of 87.7% vs 66.7% (*p* = 0.177) for the RT-sRP and FT-sRP group respectively. Postoperative functional outcomes after median follow-up of 32 months did not significantly differ between both sub-cohorts. Erectile function was comparable with mean IIEF-5-Scores of 0.5 for the RT-sRP-cohort and 2.5 for the FT-sRP-cohort (*p* = 0.199) Continence- results were also comparable with mean ICIQ-SF-Scores of 13.6 vs. 12.3 (*p* = 0.696) and continence rates of 48.4% vs 52.9% (*p* = 0.763). Detailed functional results are summarized in Table 2.

### Oncological outcomes

Kaplan–Meier survival estimates revealed no significant differences in BRFS between the RT-sRP-cohort and the FT-sRP-cohort with no significant differences in 5-year-BRFS (60% vs. 68%; *p* = 0.849) (supplementary Fig. 3).

## Discussion

The current study provides novel comparative PROM-based data on HRQOL following sRP after primary treatment with RT or FT. We found HRQOL of patients with recurrent PCa to be significantly impacted by sRP independent of the primary treatment. In line, we observed functional outcomes to be deteriorated, however cancer progression was prevented in more than 60% of all patients over a period of 5 years. These results have to be discussed in light of treatment alternatives regarding the best trade-off between HRQOL and oncological safety.

Salvage radical prostatectomy remains a challenging treatment option for radio-recurrent prostate cancer following non-surgical primary treatment. Blunt preparations, especially of posterior planes close to the rectal wall and of the neurovascular bundle are complicated due to fibrosis following radiation or focal therapy [3], with potentially negative impact on urinary continence an erectile function. To date, evidence on functional outcome following sRP remain scarce, as randomized trials are not performed in this particular area. Furthermore, HRQOL data were not included in previous studies based on cancer registries or multicenter studies [15]. Therefore, data from large prospectively maintained institutional databases is urgently required to fill this knowledge gap. Due to the scarcity of data and challenge of the procedure, sRP might be underused in daily practice. In order to counsel patients appropriately, alternative treatment options and their impact on HRQOL as well as oncological outcome have to be analyzed.

Early commencement of androgen deprivation therapy (ADT) is the standard treatment of recurrent prostate cancer [16]. ADT is known to cause toxicity, such as hot flushes, erectile dysfunction, fatigue, and gynecomastia [17]. HRQOL is thereby negatively impact by ADT [18]. In addition, especially increase of cardiovascular risk has become a concern in recent years [19]. Therefore, attempts are undertaken to avoid or shorten time of ADT exposure [20, 21]. In the setting of local recurrence, this implicates a thorough analysis of treatment alternatives and their impact on HRQOL and oncological outcomes.

Prior studies focused on the comparison of sRP to patients after primary treatment with RP. In a small case series including 13 sRP-patients after primary treatment with HIFU, Spitznagel et al. found functional outcomes to be comparable to those after RP as primary treatment. Complication rates however were more frequent after sRP [22]. In line, Nathan et al. reveal similar continence rate with 78.8% 2 years after sRP and 84.3% 2 years after RP in propensity score-matched patient cohorts [23]. Interestingly, functional outcomes for sRP were previously reported to be superior after FT compared to RT with continence rates of 77.3% versus 39.2% [7]. Our current study adds valuable insights into long-term follow-up of functional outcomes and HRQOL based on PROMs for patients who underwent sRP. Noteworthy in our study, patients already reported impaired functional data at baseline prior sRP, as 16–20% of all patients present with mild or no erectile dysfunction and only 66.7–87.5% revealed urinary continence. This rate of preoperative incontinence and erectile dysfunction in our cohort is significantly higher than the expected rate especially after FT [24]. Our study cohort might represent complex cases treated at a large referral center. Regardless of primary treatment patients reported similar erectile function with mild erectile dysfunction in 10–14.3% of all patients and a sustained continence in 48.4–52.9% of all patients.

With comparable functional outcome for both primary treatment modalities, the assessment of endpoints such as HRQOL becomes crucial for guidance of therapy. Our results revealed HRQOL to be significantly affected by salvage surgical treatment. Only about 50% of all patients in our study presented with “good general HRQOL”, defined as aGHS of ≥ 70. HRQOL remains good (GHS ≥ 70) in about 25% of all patients in long-term follow-up after salvage prostatectomy. Decline in HRQOL is markedly higher than in patients undergoing RP for primary PCa. Here, experience from our center revealed a decline of HRQOL at 3 months after the procedure but return to baseline values in the long-term follow-up [25]. HRQOL-outcomes for sRP were independent of the primary treatment in our study. This finding is especially interesting, as patients who had undergone FT that had opted for a therapy with the aim to conserve erectile function and continence [26]. Therefore, they might present with different attitude toward worsening of HRQOL or are more sensitive to changes through functional outcomes.

Regarding oncological outcome our study revealed comparable BCR-free survival rates for each primary treatment modality with a 5-year BRFS of 60% for RT and 68% for FT. In line with our findings, 5-year BCRF of 47–82% across several studies were reported in a systematic review [4]. Similar results are seen in systematic review on patients after focal therapy and sRP with a 2-year BCR-free probability of 77% [27].

This study is limited by the retrospective study design and by potential selection bias as therapy recommendation has been performed on a single-patient basis. However, as outlined in the discussion section, other study types are difficult to conduct in this setting. The retrospective single-center design focuses on open surgery and low rates of nerve-sparing might also limits the comparability in terms of functional outcomes compared to other cohorts.

## Conclusions

sRP impacts HRQOL in patients with PCa after RT and FT alike. Oncological outcomes are excellent in light of the treatment situation. As treatment alternatives including early commencement of androgen deprivation therapy are impacting HRQOL but with another profile, exploring precise patient´s preference is paramount.

## Supplementary Information

Below is the link to the electronic supplementary material.

Supplementary file 1 (DOCX 317 KB)

## Data Availability

Data is available for bonafide researchers on request from the corresponding author.

## References

[1] Marra G et al (2021) Oncological outcomes of salvage radical prostatectomy for recurrent prostate cancer in the contemporary era: a multicenter retrospective study. Urol Oncol 39(5):296.e21-296.e29 33436329 10.1016/j.urolonc.2020.11.002

[2] Preisser F et al (2023) Oncologic outcomes of lymph node dissection at salvage radical prostatectomy. Cancers (Basel) 15(12):3123 37370733 10.3390/cancers15123123 PMC10296518

[3] Calleris G et al (2019) Is it worth to perform salvage radical prostatectomy for radio-recurrent prostate cancer? A literature review. World J Urol 37(8):1469–1483 30955047 10.1007/s00345-019-02749-z

[4] Chade DC et al (2012) Cancer control and functional outcomes of salvage radical prostatectomy for radiation-recurrent prostate cancer: a systematic review of the literature. Eur Urol 61(5):961–971 22280856 10.1016/j.eururo.2012.01.022

[5] Herrera-Caceres JO et al (2020) Salvage radical prostatectomy following focal therapy: functional and oncological outcomes. BJU Int 125(4):525–530 31863617 10.1111/bju.14976

[6] Gontero P et al (2019) Salvage radical prostatectomy for recurrent prostate cancer: morbidity and functional outcomes from a large multicenter series of open versus robotic approaches. J Urol 202(4):725–731 31075058 10.1097/JU.0000000000000327

[7] Onol FF et al (2020) Comparison of outcomes of salvage robot-assisted laparoscopic prostatectomy for post-primary radiation vs focal therapy. BJU Int 125(1):103–111 31430422 10.1111/bju.14900

[8] Kretschmer A et al (2021) Health-related quality of life in patients with advanced prostate cancer: a systematic review. Eur Urol Focus 7(4):742–751 32089495 10.1016/j.euf.2020.01.017

[9] Kretschmer A et al (2015) Surgical learning curve for open radical prostatectomy: is there an end to the learning curve? World J Urol 33(11):1721–1727 25791787 10.1007/s00345-015-1540-5

[10] Aaronson NK et al (1993) The European organization for research and treatment of cancer QLQ-C30: a quality-of-life instrument for use in international clinical trials in oncology. J Natl Cancer Inst 85(5):365–376 8433390 10.1093/jnci/85.5.365

[11] Snyder CF et al (2013) Using the EORTC-QLQ-C30 in clinical practice for patient management: identifying scores requiring a clinician’s attention. Qual Life Res 22(10):2685–2691 23532341 10.1007/s11136-013-0387-8 PMC3843980

[12] Avery K et al (2004) ICIQ: a brief and robust measure for evaluating the symptoms and impact of urinary incontinence. Neurourol Urodyn 23(4):322–330 15227649 10.1002/nau.20041

[13] Rhoden EL et al (2002) The use of the simplified International Index of Erectile Function (IIEF-5) as a diagnostic tool to study the prevalence of erectile dysfunction. Int J Impot Res 14(4):245–250 12152112 10.1038/sj.ijir.3900859

[14] Pisansky TM et al (2019) Adjuvant and salvage radiotherapy after prostatectomy: ASTRO/AUA guideline amendment 2018–2019. J Urol 202(3):533–538 31042111 10.1097/JU.0000000000000295 PMC8680266

[15] Preisser F et al (2023) Impact of persistent PSA after salvage radical prostatectomy: a multicenter study. Prostate Cancer Prostatic Dis. 10.1038/s41391-023-00728-5 37803241 10.1038/s41391-023-00728-5 PMC11543598

[16] Cary KC et al (2014) Temporal trends and predictors of salvage cancer treatment after failure following radical prostatectomy or radiation therapy: an analysis from the CaPSURE registry. Cancer 120(4):507–512 24496867 10.1002/cncr.28446

[17] Nguyen PL et al (2015) Adverse effects of androgen deprivation therapy and strategies to mitigate them. Eur Urol 67(5):825–836 25097095 10.1016/j.eururo.2014.07.010

[18] Dacal K, Sereika SM, Greenspan SL (2006) Quality of life in prostate cancer patients taking androgen deprivation therapy. J Am Geriatr Soc 54(1):85–90 16420202 10.1111/j.1532-5415.2005.00567.x

[19] Rosario DJ, Bourke L (2020) Cardiovascular disease and the androgen receptor: here we go again? Eur Urol 77(2):167–169 31471137 10.1016/j.eururo.2019.08.017

[20] Nabid A et al (2018) Duration of androgen deprivation therapy in high-risk prostate cancer: a randomized phase III trial. Eur Urol 74(4):432–441 29980331 10.1016/j.eururo.2018.06.018

[21] Duchesne GM et al (2017) Health-related quality of life for immediate versus delayed androgen-deprivation therapy in patients with asymptomatic, non-curable prostate cancer (TROG 03.06 and VCOG PR 01-03 [TOAD]): a randomised, multicentre, non-blinded, phase 3 trial. Lancet Oncol 18(9):1192–1201 28760403 10.1016/S1470-2045(17)30426-6

[22] Spitznagel T et al (2021) Salvage robotic-assisted laparoscopic radical prostatectomy following focal high-intensity focused ultrasound for ISUP 2/3 cancer. Urology 156:147–153 34186136 10.1016/j.urology.2021.04.059

[23] Nathan A et al (2021) Salvage versus primary robot-assisted radical prostatectomy: a propensity-matched comparative effectiveness study from a high-volume tertiary centre. Eur Urol Open Sci 27:43–52 33997823 10.1016/j.euros.2021.03.003 PMC8090976

[24] Nicoletti R et al (2023) Functional outcomes and safety of focal therapy for prostate cancer: a systematic review on results and patient-reported outcome measures (PROMs). Prostate Cancer Prostatic Dis. 10.1038/s41391-023-00698-8 37491432 10.1038/s41391-023-00698-8

[25] Kretschmer A et al (2020) Health-related quality of life after open and robot-assisted radical prostatectomy in low- and intermediate-risk prostate cancer patients: a propensity score-matched analysis. World J Urol 38(12):3075–3083 32130477 10.1007/s00345-020-03144-9 PMC8249262

[26] Hopstaken JS et al (2022) An updated systematic review on focal therapy in localized prostate cancer: what has changed over the past 5 years? Eur Urol 81(1):5–33 34489140 10.1016/j.eururo.2021.08.005

[27] Blank F et al (2023) Salvage radical prostatectomy after primary focal ablative therapy: a systematic review and meta-analysis. Cancers 15(10):2727 37345064 10.3390/cancers15102727 PMC10216462

