# Supplementary material for: Health-related quality of life following salvage radical prostatectomy for recurrent prostate cancer after radiotherapy or focal therapy
Source: World J Urol. 2024 Apr 18;42(1):242. doi: 10.1007/s00345-024-04945-y (PMC11026200; doi:10.1007/s00345-024-04945-y)
Supplement: Supplementary file 1 — (DOCX 317 KB) [file 345_2024_4945_MOESM1_ESM.docx]

**Supplementary Files**

**Suppl. table 1:** Predictors of general HRQOL after salvage radical prostatectomy

|  | multivariate linear regression for **general HRQOL at max follow-up** | | | | |  |
| --- | --- | --- | --- | --- | --- | --- |
|  | **Variable** | **B** [regression coefficient] | **Beta** [standardized regression coefficient] | **SE** [standard error] | **p value** |  |
|  |  |  |  |  |  |  |
|  | Time between primary treatment to sRP | -0.034 | -0.064 | 0.122 | 0.788 |  |
|  | ICIQ-SF | -4.134 | -0.883 | 1.479 | ***0.021*** |  |
|  | IIEF-5 | -1.793 | -0.193 | 2.942 | 0.557 |  |
|  | Age | 1.108 | 0.386 | 0.701 | 0.148 |  |
|  | BMI | -1.291 | -0.194 | 1.676 | 0.461 |  |
|  | Prostate volume | 0.867 | 0.428 | 0.492 | 0.112 |  |
|  | R² | 0.655 | | | |  |
|  | Adjusted R² | 0.424 | | | |  |
|  |  |  |  |  |  |  |

**Supplementary Figure 1:** Patient selection
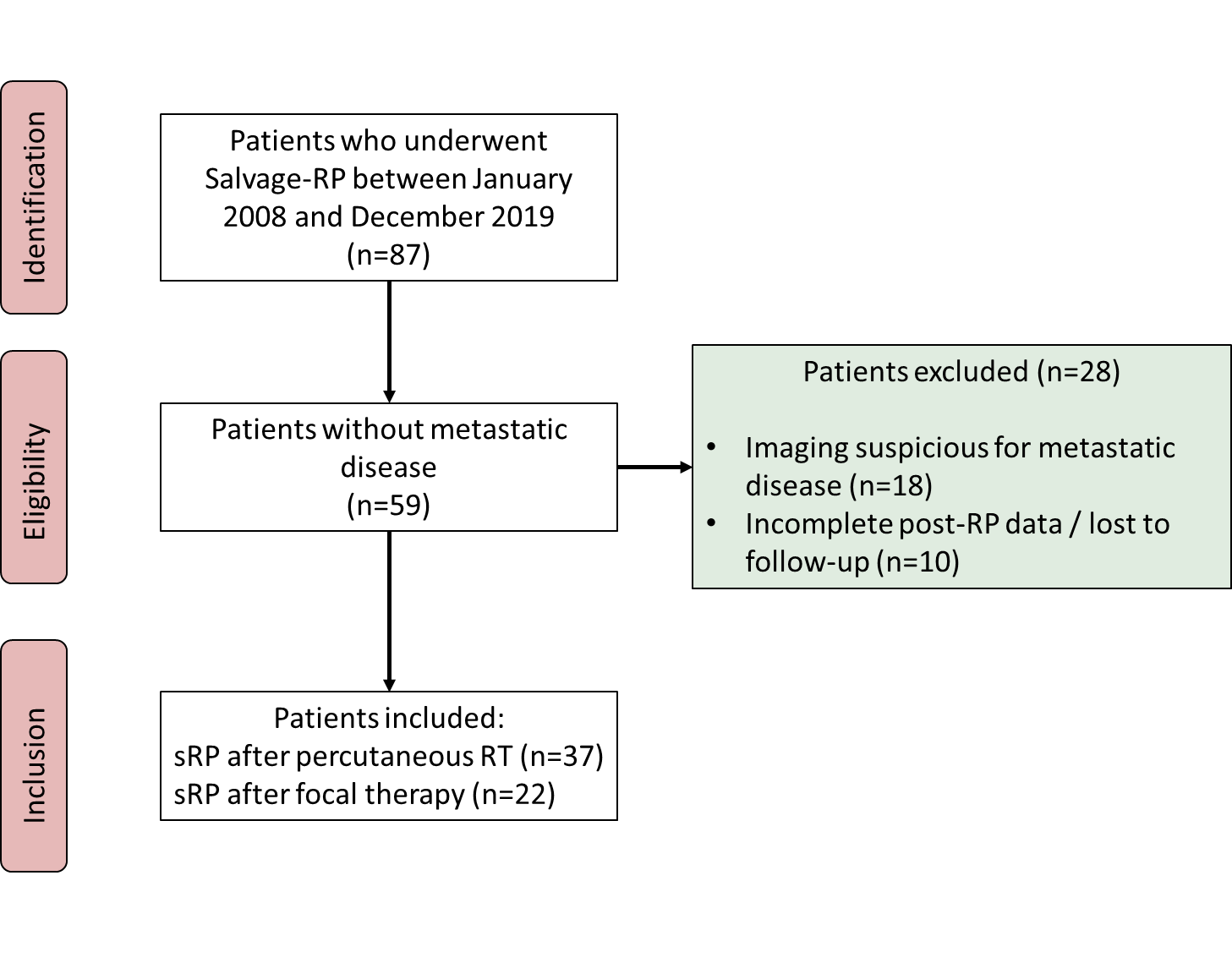


**Suppl. figure 2:** Net baseline change QLQ-C30 subscales

**
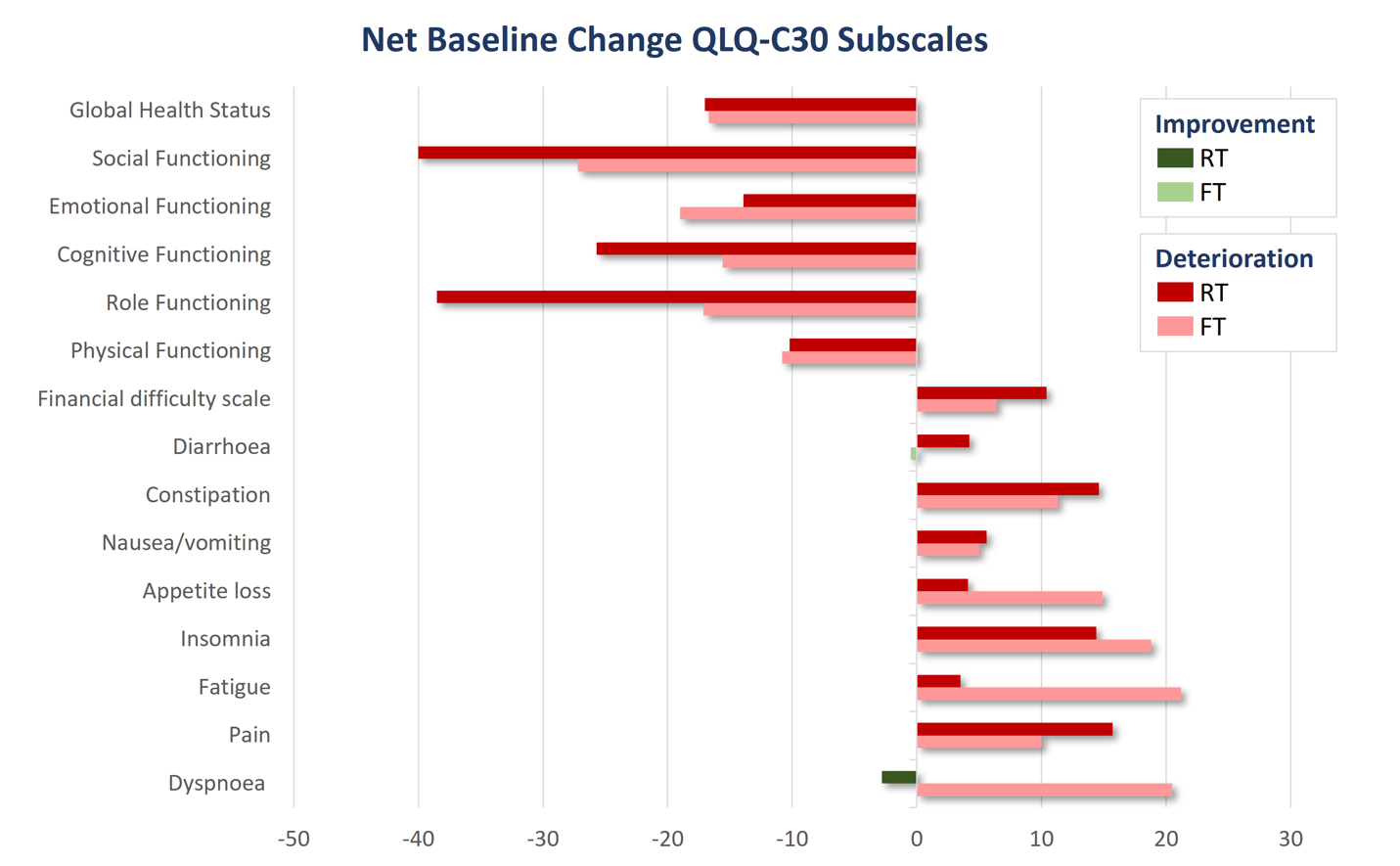
**


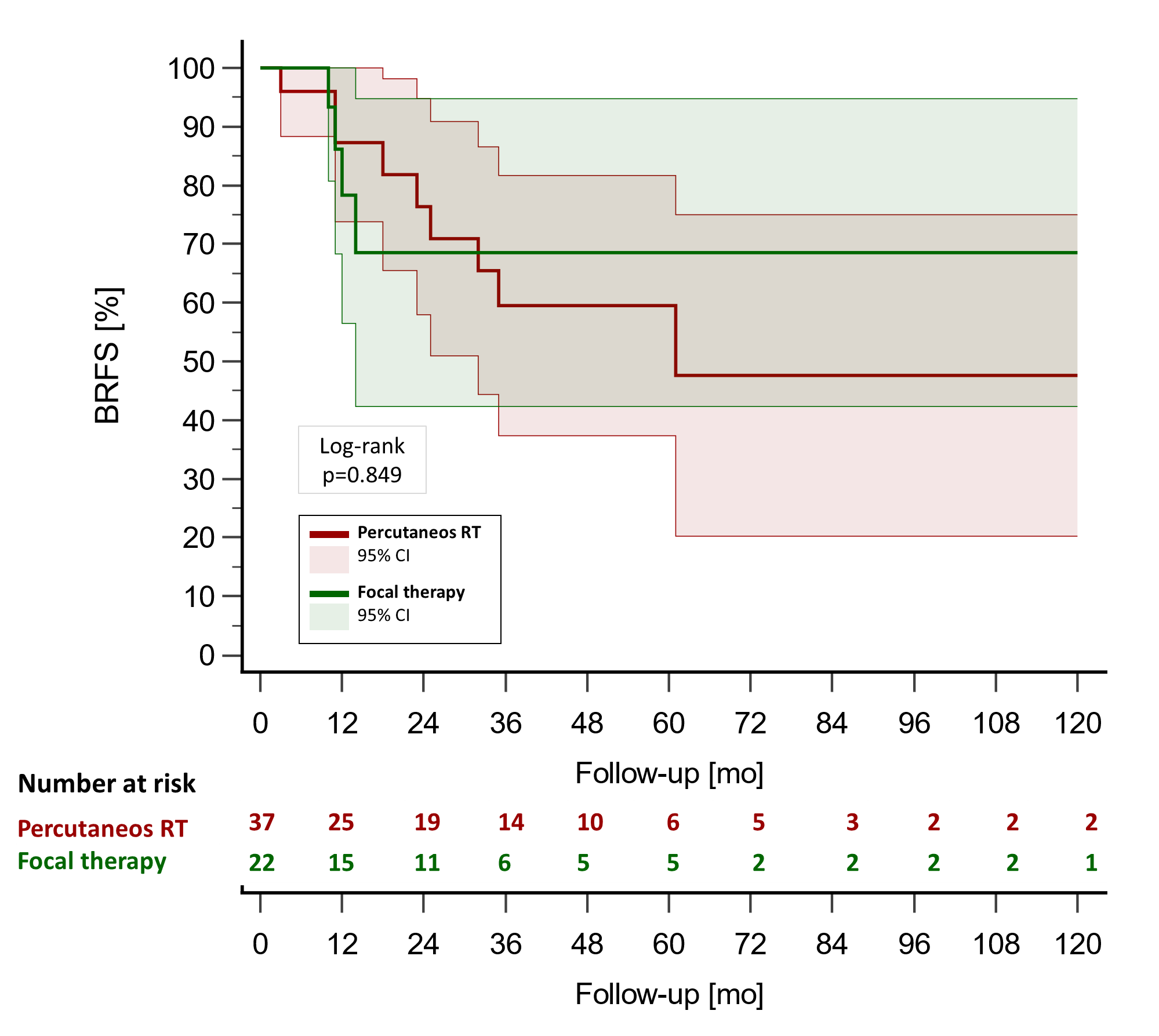
**Suppl. figure 3:** Biochemical recurrence free survival
